# Inhibition of breast cancer with transdermal tamoxifen-encapsulated lipoplex

**DOI:** 10.1186/s12951-016-0163-3

**Published:** 2016-02-19

**Authors:** Yu-Ling Lin, Chia-Hung Chen, Hsin-Yi Wu, Nu-Man Tsai, Ting-Yan Jian, Yuan-Ching Chang, Chi-Hsin Lin, Chih-Hsiung Wu, Fei-Ting Hsu, Ting Kai Leung, Kuang-Wen Liao

**Affiliations:** Department of Biological Science and Technology, National Chiao Tung University, Hsinchu, Taiwan, ROC; Center for Bioinformatics Research, National Chiao Tung University, Hsinchu, Taiwan, ROC; Institute of Molecular Medicine and Bioengineering, National Chiao Tung University, Hsinchu, Taiwan, ROC; School of Medical Laboratory and Biotechnology, Chung Shan Medical University, Taichung, Taiwan, ROC; Clinical Laboratory, Chung Shan Medical University Hospital, Taichung, Taiwan, ROC; Department of Surgery, MacKay Memorial Hospital, Taipei, Taiwan, ROC; Department of Medical Research, MacKay Memorial Hospital, New Taipei City, Taiwan, ROC; Department of Surgery, En Chu Kong Hospital, New Taipei City, Taiwan, ROC; Department of Medical Imaging, Taipei Medical University Hospital, Taipei, Taiwan, ROC; Translational Imaging Research Center, Taipei Medical University, Taipei, Taiwan, ROC; Department of Diagnostic Radiology, Taipei Medical University Hospital, Taipei, Taiwan, ROC; Graduate Institute of Biomedical Materials and Tissue Engineering, College of Biomedical Engineering, Taipei Medical University, Taipei, Taiwan, ROC; Department of Diagnostic Radiology, Taipei Hospital, Ministry of Health and Welfare, Taipei, Taiwan, ROC; College of Science and Engineering, Fu Jen Catholic University, Hsinchuang, Taiwan, ROC; Graduate Institute of Medicine, College of Medicine, Kaohsiung Medical University, Kaohsiung, Taiwan, ROC

**Keywords:** Lipo-PEG-PEI complex, Tamoxifen, Transdermal treatment, Breast cancer

## Abstract

**Background:**

Tamoxifen is currently used for the treatment of both early and advanced estrogen receptor (ER) positive breast cancer in pre- and post-menopausal women. However, using tamoxifen routinely to inhibit endogenous or exogenous estrogen effects is occasionally difficult because of its potential side effects.

**Objectives:**

The aim of this study is to design a local drug delivery system to encapsulate tamoxifen for observing their efficacy of skin penetration, drug accumulation and cancer therapy.

**Methods:**

A cationic liposome-PEG-PEI complex (LPPC) was used as a carrier for the encapsulation of tamoxifen and forming ‘LPPC/TAM’ for transdermal release. The cytotoxicity of LPPC/TAM was analyzed by MTT. The skin penetration, tumor growth inhibition and organ damages were measured in xenograft mice following transdermal treatment.

**Results:**

LPPC/TAM had an average size less than 270 nm and a zeta-potential of approximately 40 mV. LPPC/TAM displayed dramatically increased the cytotoxic activity in all breast cancer cells, especially in ER-positive breast cancer cells. In vivo, LPPC drug delivery helped the fluorescent dye penetrating across the skim and accumulating rapidly in tumor area. Administration of LPPC/TAM by transdermal route inhibited about 86 % of tumor growth in mice bearing BT474 tumors. This local treatment of LPPC/TAM did not injury skin and any organs.

**Conclusion:**

LPPC-delivery system provided a better skin penetration and drug accumulation and therapeutic efficacy. Therefore, LPPC/TAM drug delivery maybe a useful transdermal tool of drugs utilization for breast cancer therapy.

## Background

Breast cancer incidence and mortality in Taiwan was increased by approximately two to three times within nearly two decades, and Taiwanese breast cancer patients tend to be younger which is different from Western countries such as Europe and United States [1]. The age specific incidence of breast cancer in Taiwan rapidly increases in patients aged approximately 45 years. However, the equivalent peak in diagnosis occurs 5–10 years later among patients in the Western countries [2, 3]. In addition, the proliferative effects of female hormones (especially estrogen) on breast glandular cells have been proved to play the major role in the causing mechanism of breast cancer [4, 5]. In our previous study, the evidences from MRI images also revealed that overuse of estrogen or phyto-estrogen supplements can increase breast glandular tissue proliferation. Such proliferation may increase the patient’s risk of future breast cancer [3]. In Taiwan, higher incidence of breast cancer and more predominant on premenopausal age may reflect closer relationship between breast cancer and estrogenic effects, which is different to the Western countries [2].

Tamoxifen is an antagonist of the estrogen receptor via its active metabolite, hydroxytamoxifen, and is clinically administrated as the chemotherapy for estrogen receptor-positive breast cancer in pre-menopausal women [1]. Currently, Tamoxifen is also used typically for the treatment of both early and advanced estrogen receptor positive (ER+) breast cancer in pre- and post-menopausal women. For growth inhibition, Tamoxifen inhibit protein kinase C activity and ERK1/2 signaling pathway to modulate the growth of breast cancer cells [6]. Interestingly, although the ER-negative cell lines were much less sensitive to tamoxifen treatments than the ER-positive cells, Tamoxifen still caused the growth inhibition and cytotoxicity of ER-negative cell lines (such as MDA-MB-231) [7, 8]. The growth inhibition of Tamoxifen on ER-negative cell lines, are associated with a slight decrease in percentage of S-phase during cell mitosis [9]. The effects of tamoxifen-induced apoptosis in ER-negative breast cancer cells may be through inhibition of CIP2A/PP2A/p-Akt signaling pathway [10]. Thus, tamoxifen is considered as a good chemotherapeutic drug for the patients with breast cancer.

Since tamoxifen’s introduction for clinical use in the early 1970s, synthetic anti-estrogen tamoxifen citrate has been shown to contribute to controlling human breast cancer and recurrence [11]. However, the gynecologic side effects for routinely using tamoxifen are diverse and reflect the complexity of its mechanism of action. The most concerning gynecologic side effect is endometrial disease in postmenopausal women [12]. To reduce its side effect of oral administration, such as thrombosis and hepatic first-pass metabolism, as well as endometrial hyperplasia, transdermal delivery of tamoxifen using liposomal vehicle [13]. In addition, menthone in 50 % ethanol is also demonstrated it can enhance in vitro percutaneous absorption of tamoxifen [14]. Besides, Dr. Khan and his collogues showed the transdermal delivery of tamoxifen to the breast may avert the toxicity of oral tamoxifen while maintaining efficacy in Phase II presurgical trial [15]. However, decrease in 3.4 % of Ki67 after therapy in the previous study in which the delivery efficiency is considered it can be improved.

The cationic liposome-PEG-PEI complex (LPPC) had been developed for encapsulating drug and protected drugs’ structural stability [16–18]. LPPC helps the drugs rapidly penetrate into cell plasma to result in an excellent therapeutic effect in vitro and in vivo [17, 18]. Because LPPC can strongly capture antigens or immunomodulators onto its surface, LPPC has also been used as an adjuvant and triggered the Th2 immune responses and antibodies class switch [19]. These results therefore suggest that LPPC may serve as an effective drug carrier and a useful anticancer tool.

In this study, LPPC was designed as a local drug delivery system for tamoxifen encapsulation and to observe their efficacy of skin penetration and therapeutic efficiency. LPPC/TAM has higher cytotoxicity in all breast cancer cell lines. In vivo studies showed that LPPC/TAM can efficiently penetrate across the skins of the mice and increase the drug accumulation in tumor. LPPC/TAM also significantly inhibit the tumor growth of the BT474 tumor. Therefore, the LPPC/TAM is worthy to further develop as an anti-breast cancer drug by transdermal treatment.

## Results

### The characteristics of LPPC/TAM

**Fig. 1:**
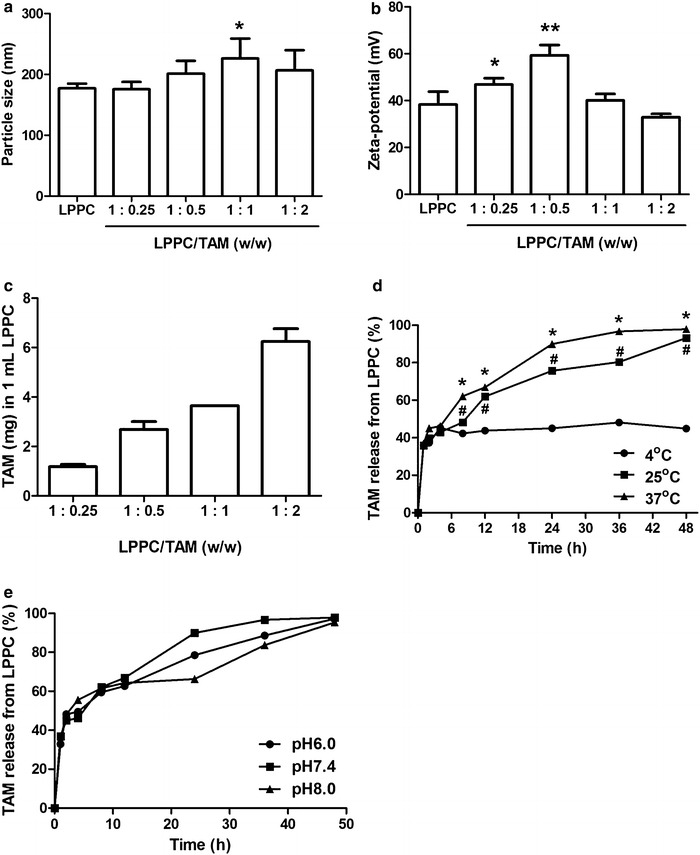
The characteristics of LPPC/TAM. TAM encapsulated in 10 mg of LPPC at various weight ratios. The (**a**) particle size, (**b**) zeta-potential and (**c**) TAM encapsulation of LPPC/TAM were measured. *p < 0.05 or ** *p* < 0.01, compared with the LPPC group. **d** TAM release from LPPC/TAM in PBS at 4, 25 and 37 °C. *p < 0.05, 37 °C group was compared with the 4 °C group; ^#^ *p* < 0.05, 25 °C group was compared with the 4 °C group. **e** TAM release from LPPC/TAM in PBS with pH 6.0, 7.4 or 8.0 at 37 °C. After the incubation, the percentage of TAM in each supernatant was measured and compared with the total amount of TAM (n = 6)

The particle sizes of different LPPC/TAM mixtures (weight ratios of LPPC/TAM from 1:0.25 to 1:2) ranged from 180 to 230 nm (Fig. 1a). The average zeta-potential of these different LPPC/TAM mixtures ranged from 38 to 60 mV (Fig. 1b). For TAM encapsulation, the maximal encapsulation capacity of 1 mg of LPPC was ~6200 µg of TAM at 1:2 (w/w) ratios of LPPC/TAM (Fig. 1c). According to the results of particle size, zeta-potential, and BP encapsulation capacity assessments, the 1:2 ratio of LPPC/TAM was chosen for the following experiments.

To understand drug release from LPPC/TAM complexes, in vitro drug release was determined. The kinetics of drug release showed that only 35–42 % of the encapsulated TAM was released from LPPC/TAM after incubation at 4 °C (Fig. 1d). Approximately 90 and 98 % of the encapsulated TAM had been released into the media after 48 h-incubation at 25 and 37 °C. When LPPC/TAM was incubated in PBS solution with different pH value at 37 °C, the kinetics of TAM release was similar (Fig. 1e).

**Fig. 2:**
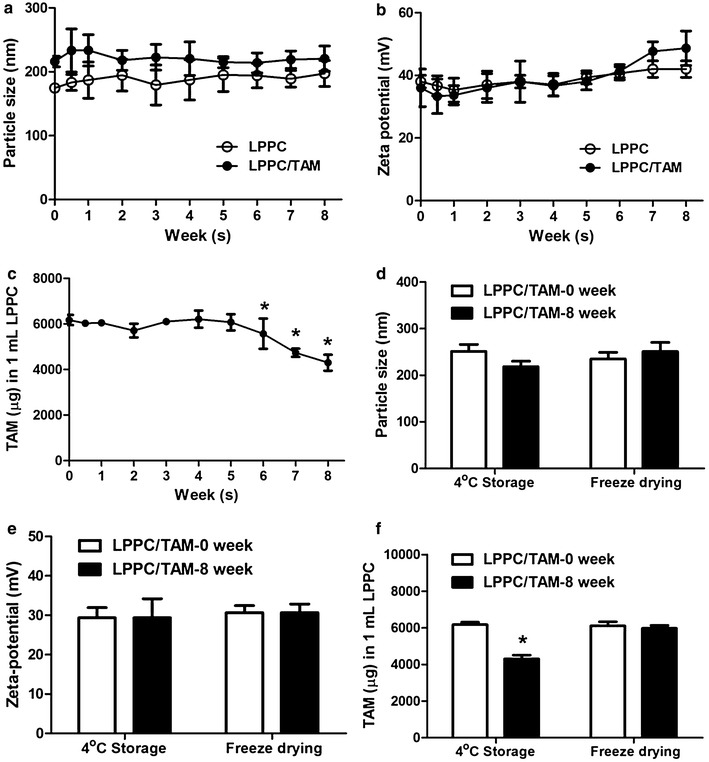
Stability of LPPC/TAM. LPPCs and LPPC/TAMs were storage at 4 °C for 8 weeks. **a** Particle size, **b** zeta-potential and **c** TAM amount in LPPC were measured at different storage time (n = 6). LPPC/TAMs were processed by freeze drying and storage at 4 °C for 8 weeks. **d** Particle size, **e** zeta-potential and **f** TAM amount in LPPC were measured (n = 3). *p < 0.05, compared with the 0 week

Moreover, the stability of LPPC/TAM was investigated for further analysis and application. Figure 2a, b indicated that the particle sizes and zeta-potentials did not significantly change at 8 weeks as well as fresh preparation (0 week). Over 90 % of TAM retained in LPPC/TAM complexes within 6 weeks, even 70 % of TAM retained at 8 week (Fig. 2c). Since drug storage is an important issue for the future clinical application, LPPC/TAM were processed by freeze drying for storage. After 8 weeks incubation, freeze dried LPPC/TAM were rehydrated with DDW, and then the particle sizes, zeta-potentials and TAM contents in LPPC were measured. Figure 2d, e showed that the characteristics of freeze dried LPPC/TAM were similar to fresh prepared LPPC/TAM and TAM was stably kept in LPPC within 8 weeks.

### The cytotoxic activity of LPPC/TAM against breast cancer cells

**Fig. 3:**
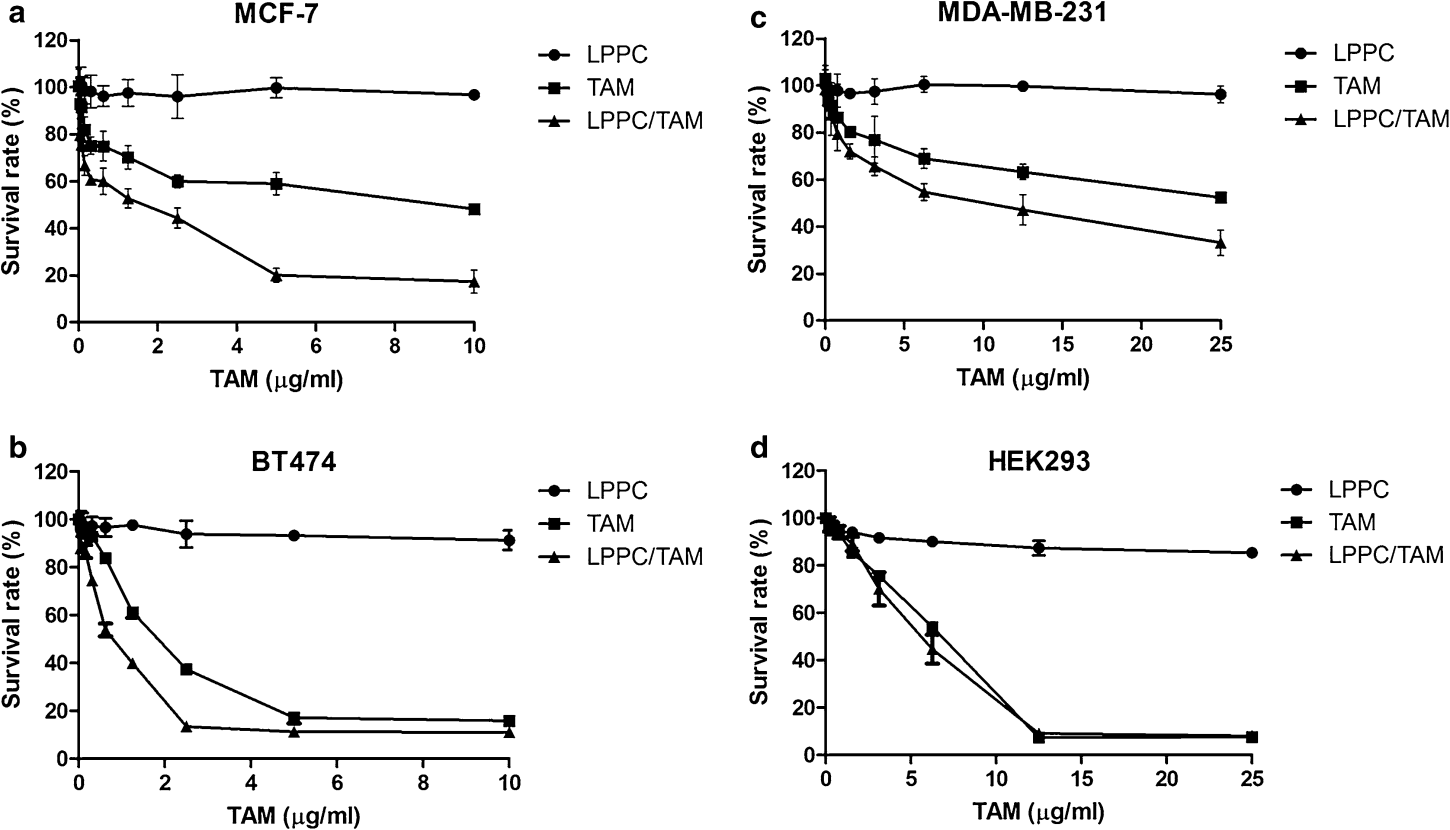
Cytotoxicity of LPPC-TAM in breast cancer cells. **a** MCF-7 (ER+), **b** BT474 (ER+), **c** MDA-MB-231 (ER−) and **d** human embryonic kidney HEK293 cells were treated with different concentrations LPPC, TAM or LPPC/TAM for 72 h. The cell viability was analyzed by MTT cytotoxicity (n = 6)

To determine whether LPPC/TAM could enhance the cytotoxicity of TAM in different types of breast cancer cells, such as ER-positive MCF-7 and BT474 cells and ER-negative MDA-MB-231 cells were analyzed by MTT assay. The results of Fig. 3a, b indicated that the LPPC complexes enhanced the cytotoxic effects of TAM on ER+ cells, such as MCF-7 and BT474 cells. Interestingly, LPPC also increased the cytotoxic effects of TAM on MDA-MB-231 (ER−) cells (Fig. 3c). The cytotoxicity of LPPC/TAM was similar to TAM in HEK293 cells (Fig. 3d).

### Transdermal LPPC/DiI absorption in vivo

**Fig. 4:**
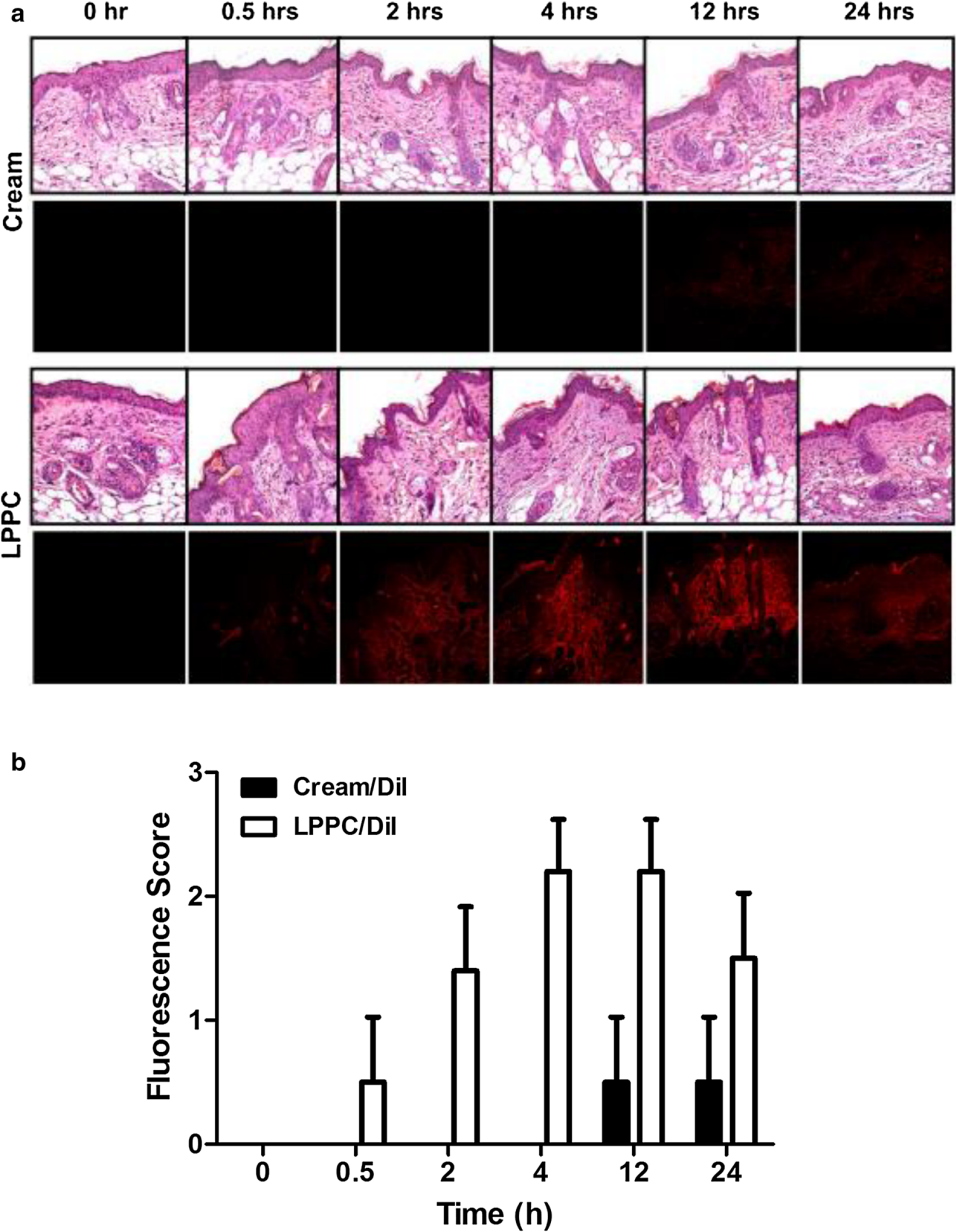
In vivo skin permeation of LPPC/DiI or cream/DiI after treatment in BT474 tumor-bearing mice. **a** BT474 tumor-bearing mice were applied the cream and LPPC containing DiI fluorescent dye (*red fluorescence*) to tumor area once. The tumor sample covered with skin were collected and fixed at different time periods. All fixed tumor samples were vertically cut and the fluorescent intensity was evaluated for permeation efficiency by fluorescent microscope. Hematoxylin & Eosin staining in *above panels* showed the structure of skin to tumor. *Below panel* showed the drug accumulation by skin permeation. **b** The DiI accumulation in tumor area after treatment with LPPC/DiI or cream/DiI. The fluorescent score were identified as 0–3 for <5, 5–25, 26–50 and >50 %, respectively

This study examined the effect of LPPC encapsulation of drug on the permeability in transdermal drug delivery using the DiI as a florescent probe. The results showed that the permeability of cream/DiI were detectable after 12 h. However, LPPC/DiI significantly facilitated the passage of drug through skin after treatment for 30 min (Fig. 4a). In tumor area, LPPC enhanced a large number of DiI accumulated after 2 h treatment, while the <5 % DiI accumulation in the cream/DiI group (Fig. 4b).

### Effects of LPPC/TAM on tumor growth inhibition and organs damage

**Fig. 5:**
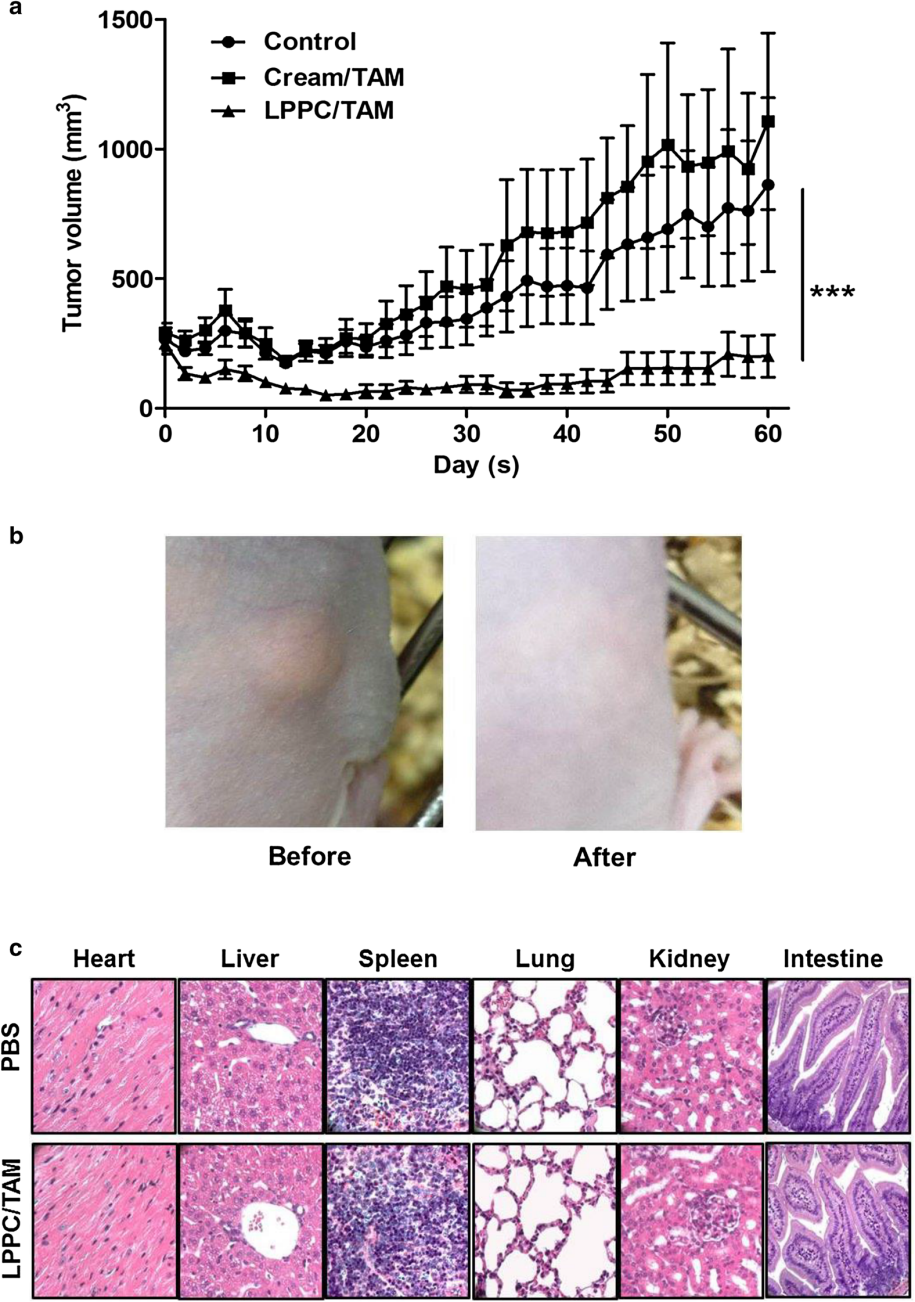
Anti-tumor effects of LPPC/TAM by transdermal treatment. **a** BT474 tumor-bearing mice were applied the cream/TAM or LPPC/TAM to tumor area every day. Tumor volume was measured with a caliper, and tumor volume was calculated as L × H × W × 0.5236. The animals were sacrificed over 60 days after implantation of the 60-d release 17β-estradiol pellet (n = 5). **b** Observations of the skin in the tumor bearing mouse before or after treatment with LPPC/TAM. **c** Histopathological features of the hearts, livers, spleens, lungs, kidneys and intestine in the LPPC/TAM treatment group. Portions of the organs were fixed in 10 % formaldehyde overnight, embedded in paraffin and cut into slices. Organ sections were stained with H and E

Athymic mice bearing BT474 tumors were treated with cream/TAM (25 mg/kg), empty LPPC or LPPC/TAM (containing 25 mg/kg TAM) every day by application. The animals treated with LPPC/TAM showed a significant suppression of BT474 tumor growth compared with the control group by approximately 82 %, even two mice with tumor had been cured (Fig. 5a). These results also showed that LPPC/TAM was more effective than cream/TAM on inhibiting BT474 tumor growth. Additionally, to evaluate the tissue damage induced by LPPC/TAM treatment, pathology of organs had been analyzed. Figure 5b showed that LPPC/TAM did not hurt any organs and induce any irritation of the skin in the treated mice (Fig. 5c). Therefore, this local treatment with LPPC/TAM maybe a safe and efficient breast cancer therapeutics.

## Discussion

Transdermal drug delivery system offers sustainable release of drugs in local area. However, permeation of the most of the drugs across the skin barrier remains a major limitation. In this study, LPPC/TAM successfully passed through in vivo animal skin, helped drugs accumulation in subcutaneous tumor (Fig. 4) and efficiently inhibited the tumor growth (Fig. 5). LPPC/TAM significantly increased the inhibition of subcutaneous tumor growth by transdermal treatment due to positive charge on its surface. These cationic liposomes, which are promising carriers for transdermal treatment, have been found to efficiently pass the skin cells into tumors [20, 21]. Thus, LPPC/TAM, a cationic polymer-liposome composed PEI, helped drug rapidly across skin and accumulated in tumor area. In addition, drug-loaded LPPC should be able to release the drug in the tumor area in a stable (Fig. 1) and controlled manner [17]. Therefore, LPPC should give rise to the highly efficient penetration of the drug into tumor area.

LPPC provide an advanced encapsulation that was able to efficiently deliver TAM into tumor cells. When compared with nonencapsulated TAM, LPPC/TAM not only dramatically increased the cytotoxic activity of TAM from 6.7- to 7.9-fold in all breast cancer cells in vitro (Fig. 3), but was also able to inhibit 82 % of subcutaneous tumor growth in vivo by transdermal treatment (Fig. 5). In our previous report found it that LPPC encapsulation could also suppress the cell proliferation of drug-resistant cells and elevate the anti-proliferative effect of drug [22]. The increased antiproliferative effect of LPPC/TAM may be due to its increased ability to rapid penetrate and accumulate in cells. This high level of efficient transport into cells is supported by the fact that LPPC is a good shuttle carrier for drugs across the cell membrane. LPPC has been shown to be an excellent drug transporter, capable of delivering large quantities of encapsulated molecules across the cell membrane quickly, which would likely explain the benefits observed by LPPC/TAM treatment in all breast cancer cells containing ER-positive and ER-negative cells.

Transdermal drug delivery systems decrease side effects and prevent drug from metabolism by systemic treatment. Tamoxifen had been approved by FDA for breast cancer risk reduction and currently used for the treatment of both early and advanced ER positive breast cancer. However, the side effects of tamoxifen, particularly endometrial cancer and thromboembolic complications, have limited the drug’s uptake by high-risk women who should benefit from its preventive effects [12, 23]. In addition, breast cancer patients have CYP2D6 polymorphisms associated with loss of its enzyme function for tamoxifen metabolism [24]. CYP2D6 is a key enzyme for the biotransformation of tamoxifen into endoxifen which has more binding activity of ER than tamoxifen [24]. Breast cancer patients with lower CYP2D6 activity caused of their genotype produce less endoxifen and lead to inferior therapeutic benefit from tamoxifen [25, 26]. Therefore, various tamoxifen formulations had been developed for solving side effects or metabolism problems of tamoxifen [27–31]. In clinical trials, tamoxifen has already modified the appropriate formulations as 4-hydroxytamoxifen gel which successfully penetrates the skin to reach breast tissue for a preventive effect [15, 32]. However, the gel formulation of tamoxifen less penetrated at 24 h across skin was similar to that in low penetrable liposomal vesicles was limited to upper epidermis layer [13, 32]. In our study, LPPC transdermal drug delivery system allowed drug rapidly and deeply penetration at 2 h (Fig. 4) and more therapeutic effects of tamoxifen (Fig. 5). Thus, transdermal treatment of LPPC/TAM might provide the breast cancer patients with CYP2D6 polymorphisms another efficient therapeutics and avoid the side effects of systemic treatment under tamoxifen administration.

## Conclusion

We believe that the application of LPPC/TAM as a transdermal treatment have significant potential for preventing the estrogen-induced proliferative effect on breast cancer cells that alleviating future breast cancer development and progression.

## Methods

### Lipo-PEG-PEI complex for encapsulating tamoxifen (LPPC/TAM)

LPPCs were prepared following a previously described protocol [33]. For LPPC/TAM preparation, different weight ratios of TAM (AstraZeneca, London, UK) and 1 mg of LPPC were vigorously mixed for 15 s and incubated for 30 min. After incubation, the mixture of tamoxifen and LPPC were centrifuged at 5900× *g* for 5 min to remove the free tamoxifen in the supernatant. The pellets (LPPC/TAM) were resuspended with 3 ml phosphate-buffered saline (PBS) buffer for following studies.

### Particle size and zeta-potential of LPPC/TAM

The particle sizes and zeta potentials of the empty LPPC and LPPC/TAM were determined using a Zetasizer instrument (Zetasizer 3000HS, Malvern Instruments, Malvern, UK). To further determine the stability of LPPC/TAM complexes, the TAM of encapsulated capacity, size and zeta potential were also evaluated every week.

### TAM encapsulation in LPPC/TAM

To measure the amount of tamoxifen in LPPC, LPPC/TAMs were destroyed by chloroform and added the distilled water (DDW) for partition. The concentration of TAM remaining in the chloroform layer was then measured using a UV spectrophotometer (Amersham Biosciences, Uppsala, Sweden) at 300 nm.

### In vitro drug release from LPPC/TAM

The in vitro TAM release from LPPC/TAM complexes were evaluated using a dialysis bag diffusion technique. LPPC/TAM complexes were suspended in 1 ml of PBS and then placed into a dialysis bag (Spectra/Por, Spectrum Laboratories Inc., Rancho Dominguez, California) with a 6–8 kDa molecular weight cutoff and was immersed into 500 ml of PBS at 4, 25 or 37 °C with continuous stirring. LPPC/TAM also incubated in PBS with pH 6.0, 7.4 or 8.0 at 37 °C for measuring the drug release. Then 1 ml of the sample was collected from the incubation medium and measured for TAM concentration as described above. The release rate was calculated as follows:

$${\text{Release rate }}\left( \% \right) \, = \, \left( {\text{Released TAM}} \right){\text{ amount}}/ \, \left( {\text{Total TAM in LPPC}} \right){\text{ amount }} \times { 1}00 \%.$$

### Cells and culture conditions

Human breast cancer cell lines were such as MDA-MB-231, MCF-7 and BT474 cells and human embryonic kidney 293 cells (HEK293) were purchased from the Bioresources Collection and Research Center (Hsinchu, Taiwan). All cells were grown in a humidified atmosphere with 5 % CO2 at 37 °C and subcultured with a 0.1 % trypsin, 2 mM EDTA solution. MDA-MB-231, MCF-7 and HEK293 cells were maintained in Dulbecco’s Modified Eagle’s medium (Invitrogen Carlsbad, CA) and BT474 maintained in HybriCare medium (Invitrogen) supplemented with 10 % heat inactivated fetal bovine serum (Gibco BRL, Gaithersburg, USA) and 1 % Penicillin/streptomycin (PS, Biological industries, Beithaemek, Israel).

### Cytotoxicity of LPPC/TAM

Cells were seeded in 96-well tissue culture plates at a concentration of 1 × 10^4^ cells/100 μl/well overnight. Subsequently, the cells were treated with serial concentrations of the various agents, such as empty LPPC, LPPC/TAM and non-encapsulated TAM. After 72 h of incubation, the levels of cell viability for each cell line were determined by MTT colorimetric assay. The cell viability values were plotted as a percentage of the untreated control. Briefly, 100 µL of 2 mg/mL MTT reagent (Sigma-Aldrich, St Louis, MO, USA) was added to each well, and the solution was allowed to incubate for 5 h at 37 °C. Then, the media was aspirated, and 100 µL of dimethyl sulfoxide was added to each well. Finally, the OD_595_ of each well was measured by an ELISA reader (Tecan, Mannedorf, Switzerland). Cell viability was plotted as a percentage of the untreated control.

### In vivo skin permeation of LPPC-DiI and cream-DiI

Female athymic mice (4–6 weeks of age) were obtained from the National Laboratory Animal Center (Taipei, Taiwan). All procedures were conducted in compliance with the standard operating procedures of the Laboratory Animal Center of National Chiao Tung University (Hsinchu, Taiwan). Nude mice (n = 5) were implanted s.c. with 2 × 10^7^ BT474 cells suspended in a 50 μL mixture of PBS and Matrigel Matrix High Concentration (BD Biosciences, Mountain View, California) at a 1:1 ratio after implantation with the 60-d released 17β-estradiol pellet (Innovative Research of America, Sarasota, FL). BT474 tumor-bearing mice were plastered the cream and LPPC containing DiI fluorescent dye (red fluorescence) to tumor area until absorption. The tumor sample covered with skin were collected and fixed at different time periods. All fixed tumor samples were vertically cut and the fluorescent intensity was evaluated for permeation efficiency by the fluorescent microscope.

### Anti-tumor activity of TAM/LPPC

Athymic mice (n = 5) bearing BT474 tumor were treated with cream/TAM (containing 25 mg/kg TAM), TAM/LPPC (containing 25 mg/kg TAM) or vehicle by skin applied every day after tumor establishment. Tumor size was measured with a caliper, and tumor volume was calculated as L × H × W × 0.5236. The animals were sacrificed over 60 days after implantation with the 60-d released 17β-estradiol pellet.

### Tissue damage in organs

After treatment with TAM/LPPC, the mice were sacrificed. Paraffin-embedded sections were obtained from the organs such as heart, liver, spleen, lung, kidney and intestine treated with LPPC/TAM by transdermal treatment were processed for H and E staining. Tissue morphology was observed under a microscope (Olympus, Center Valley, PA, USA).

### Statistical analysis

All analyses were performed using SPSS statistical software. Statistical evaluations of data were performed using a *t* test. Datasets with a *P* value of <0.05 were considered significant.
